# IgA Nephropathy: Significance of IgA1-Containing Immune Complexes in Clinical Settings

**DOI:** 10.3390/jcm13154495

**Published:** 2024-08-01

**Authors:** Hitoshi Suzuki, Jan Novak

**Affiliations:** 1 Department of Nephrology, Juntendo University Urayasu Hospital, 2-1-1 Tomioka, Urayasu 279-0021, Chiba, Japan; 2 Department of Microbiology, University of Alabama at Birmingham, Birmingham, AL 35294, USA

**Keywords:** IgA nephropathy, galactose-deficient IgA1, immune complexes, biomarker

## Abstract

IgA nephropathy (IgAN) is considered to be an autoimmune disease characterized by the formation of IgA1-containing immune complexes in the circulation and glomerular immunodeposits. Extensive research has identified multiple genetic, immunological, and environmental factors contributing to disease development and progression. The pathogenesis of IgAN is considered a multifactorial process involving the formation of immune complexes wherein aberrantly *O*-glycosylated IgA1 is recognized as an autoantigen. Consequently, the clinical presentation of IgAN is highly variable, with a wide spectrum of manifestations ranging from isolated microscopic hematuria or episodic macroscopic hematuria to nephrotic-range proteinuria. Whereas some patients may exhibit a slowly progressive form of IgAN, others may present with a rapidly progressive glomerulonephritis leading to kidney failure. Development of the treatment for IgAN requires an understanding of the characteristics of the pathogenic IgA1-containing immune complexes that enter the glomerular mesangium and induce kidney injury. However, not all details of the mechanisms involved in the production of galactose-deficient IgA1 and immune-complex formation are fully understood. Here, we review what we have learned about the characteristics of nephritogenic IgA1 in the half-century since the first description of IgAN in 1968.

## 1. Introduction: Diagnosis and Limitation of Risk Assessment in IgAN

IgA nephropathy (IgAN) is defined by mesangioproliferative glomerulonephritis with glomerular deposition of IgA-containing immune complexes in the mesangial regions. Up to 40% of patients progress to kidney failure within 20 years after diagnosis [1]. Outcomes of IgAN patients are generally poor, with only a few patients expected to avoid kidney failure in their lifetime. Significantly, patients traditionally regarded as being low risk, with proteinuria <0.88 g/g creatinine, had high rates of kidney failure within 10 years [2]. The poor prognosis of IgAN is partly due to the delayed diagnosis because of a lack of specific biomarkers. Currently, pathological assessment of renal biopsy specimens remains the gold standard for a definitive diagnosis of IgAN. By routine immunofluorescence, complement C3 is usually detected along with IgA and often with co-deposition of IgG and/or IgM [1]. The renal biopsy evaluation of IgAN biopsy specimens is useful to assess disease activity, chronicity, and prognosis. IgA deposition, together with IgG/IgM, may be associated with disease activity [3]. Co-deposition of IgG is not always detected in the kidney biopsy specimens of IgAN patients by routine immunofluorescence microscopy. A nanobody specific for the CH3 domain of its Fc portion detects IgG in all patients with IgAN [4]. Renal biopsy reporting should provide MEST-C scores based on the Oxford classification. The Oxford classification is composed of the pathological findings of mesangial hypercellularity (M), endocapillary hypercellularity (E), segmental glomerulosclerosis (S), tubular atrophy/interstitial fibrosis (T), and cellular/fibrocellular crescent formation (C). Especially T and C scores are predictive of poor renal outcomes [5]. However, differences in the time of renal biopsy from disease onset may yield variable pathological severity and chronicity. Pathological findings may show a combination of acute inflammatory lesions and chronic lesions according to the clinical course. Thus, renal biopsy evaluation is an assessment of pathological changes at a specific time and has an inherent limitation for the assessment of disease activity. Moreover, there are different indications for renal biopsy in different countries [6]. Here, we review the current understanding of the pathophysiology of IgA1-immune-complex formation in IgAN and the implications for clinical practice.

## 2. Approaches for Assessment of Urinary Abnormalities in IgAN

Several clinical factors have been identified that are associated with disease progression, such as sustained proteinuria over a gram per day, baseline estimated glomerular filtration rate (eGFR), change in eGFR over time, high blood pressure, and histopathological severity [7]. The degree of proteinuria is one of the most important prognostic factors, not only for IgAN but also for other renal diseases [8], and a decrease in kidney function and a reduction in proteinuria have been considered endpoints in clinical studies of renal diseases. Consequently, clinical guidelines often recommend therapeutic approaches based on the level of proteinuria. A difficulty is in distinguishing proteinuria due to acute glomerular inflammatory lesions vs. chronic lesions. IgAN tends to have a long chronic clinical course with variable combinations of acute inflammatory lesions and the common pathways of chronic lesions. For these reasons, it is difficult to discriminate the acute phase vs. chronic phase based on a qualitative assessment of proteinuria, thus limiting the utility of proteinuria for the assessment of disease activity.

Most IgAN patients show microscopic hematuria with varying degrees of proteinuria. Hematuria in IgAN is of glomerular origin, resulting from chronic inflammation elicited by IgA1-containing immune complexes and the resulting mesangial-cell activation, mesangio-podocytic crosstalk, and endothelial injury compromising the barriers retaining red blood cells in the capillaries. Macroscopic hematuria often occurs during or after upper-respiratory tract infections, suggesting that glomerular hematuria is associated with disease activity in IgAN [9,10]. In fact, IgAN patients with consistently elevated time-averaged hematuria had a significantly greater decrease in eGFR when compared to their counterparts who had negligible hematuria [11]. Recently, trajectory patterns stratified by the magnitude of hematuria and proteinuria using a nationwide multicenter chronic kidney disease registry in Japan identified high-risk IgAN patients [9]. However, the degree of hematuria may also be caused by urinary infections, urinary stones, and malignancies. Taken together, additional methods are needed for the assessment of disease activity in addition to renal biopsy and urinalysis.

## 3. Pathophysiology of Formation of Immune Complexes Containing Galactose-Deficient IgA1 

**Figure 1:**
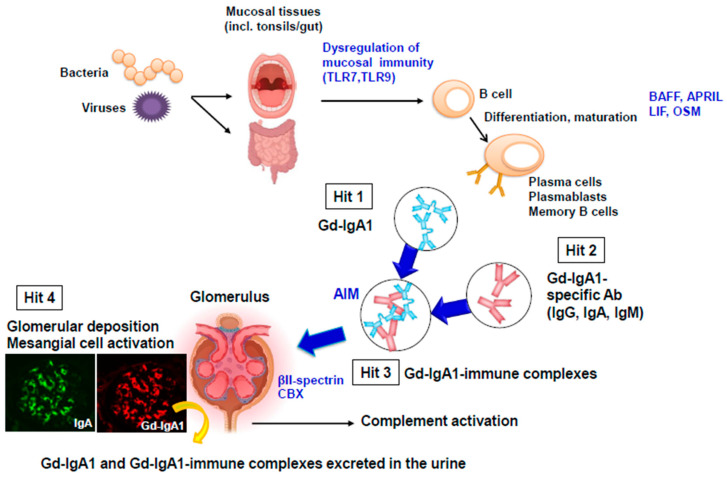
Mechanisms contributing to the production of Gd-IgA1 and the formation of pathogenic immune complexes. Mucosal immune dysregulation in NALT and/or GALT is thought to be involved in the production of Gd-IgA1. TLR9 and TLR7 play a crucial role in the overproduction of Gd-IgA1 via plasma-cell differentiation that results in secretion of Gd-IgA1 or autoantibodies specific for Gd-IgA1 that drive the formation of Gd-IgA1-containing immune complexes. Some of these complexes deposit in the glomeruli, activate mesangial cells, and induce kidney injury through complement activation. The Gd-IgA1-driven multi-hit process is considered the most likely mechanism for the pathogenesis of IgAN. LIF; leukemia inhibitory factor, OSM; oncostatin, AIM; apoptosis inhibitor of macrophages. CBX; chromobox homolog.

Galactose-deficient IgA1 (Gd-IgA1) is the key molecule involved in the pathogenesis of IgAN [12]. Macroscopic hematuria, concurrent with or appearing after upper-respiratory tract infections or gastroenteritis, is a common manifestation of IgAN. Dysregulation of the mucosal immune system, namely the tonsils in the nasal-associated lymphoid tissue (NALT), is thought to be involved in the production of Gd-IgA1 [13]. Autoantibodies specific for Gd-IgA1 bind to Gd-IgA1, thus driving the formation of circulating immune complexes, with some additional proteins being added to these complexes. Some of these complexes deposit in the glomeruli, activate mesangial cells, and induce kidney injury. The Gd-IgA1-driven multi-hit process is considered the most likely mechanism for the pathogenesis of IgAN [1] (Figure 1). The pathogenic potential of Gd-IgA1-IgG immune complexes was confirmed *in vivo* using mice injected with pre-formed immune complexes [14].

## 4. Dysregulation of Mucosal Immunity

One of the important clinical features of IgAN is that macroscopic hematuria often appears during or shortly after a mucosal infection of the upper-respiratory tract. This synpharyngitic hematuria is not limited to a specific viral or bacterial infection. These infections may thus trigger innate as well as adaptive immune responses. There is mounting evidence suggesting that the activation of Toll-like receptors (TLRs) is involved in the pathogenesis of IgAN. TLRs are receptors recognizing pathogen-associated molecular patterns and, as such, may be involved in the pathogenesis of various autoimmune diseases, including IgAN [15,16]. Among the TLRs, single-nucleotide polymorphisms of TLR9, the receptor that recognizes microbial unmethylated CpG DNA, were associated with the pathological severity of IgAN [17]. In a murine IgAN model, TLR9 activation enhances serum levels of aberrantly glycosylated IgA and IgG-IgA immune complexes, consequently leading to the development of glomerular IgA deposits [18].

Mucosal infections also lead to the production of cytokines and growth factors that can impact B cells and their biology. For example, IL-6 can downregulate the expression of core 1 β1,3-galactosyltransferase (C1GalT1) and core 1 β3-Gal-T-specific molecular chaperone (Cosmc), resulting in the overproduction of Gd-IgA1 [19]. APRIL is a member of the tumor necrosis factor superfamily and plays a key role in B-cell maturation and IgA-class switch [20]. Expression of APRIL and its receptors in human tonsils are elevated in patients with IgAN, and the tonsillar expression of TLR9 and APRIL correlates with the treatment response to tonsillectomy [21]. Notably, TLR9 and APRIL pathways interact, wherein TLR9 activation leads to the production of aberrantly glycosylated IgA via IL-6- and APRIL-mediated pathways [22].

TLR7, a pattern-recognition receptor that recognizes microbial single-stranded RNA, can also enhance the production of Gd-IgA1 and thus impact disease progression in IgAN [23]. In addition to TLR7, TLR9, another of the nucleotide-sensing TLRs, is also expressed in the endosomes of B cells, dendritic cells, and macrophages [24]. Notably, the activation of TLR7 accelerates the disease progression of IgAN [25]. Moreover, the activation of either TLR9 or TLR7 promotes the synthesis of aberrantly glycosylated IgA [25]. Thus, the activation of TLR9 and TLR7 in mucosa might be involved in the overproduction of Gd-IgA1, leading to enhanced immune-complex formation and glomerular deposition (Figure 1).

In agreement with this hypothesis, several clinical trials determined that hydroxychloroquine (HCQ), an inhibitor of TLR signaling, effectively reduces proteinuria in patients with IgAN [26]. HCQ is widely used in autoimmune diseases such as systemic lupus erythematosus and rheumatoid arthritis, and its immunomodulatory effects include the suppression of inflammatory cells, the inhibition of autoantigen presentation, and the inhibition of TLRs and cytokine signaling [27]. HCQ accumulation in the lysosomes and autophagosomes increases the organelles’ pH and thus inhibits MHC-II antigen presentation and the subsequent differentiation and activation of T and B cells. HCQ also inhibits the production of some antiviral interferons [28]. In a murine IgAN model, HCQ administration reduced kidney injury in ddY mice [25], although the mechanism of its activity in IgAN is not well understood.

## 5. Leukemia Inhibitory Factor and Its Involvement in Gd-IgA1 Synthesis

Genome-wide association studies (GWASs) have identified several susceptibility loci associated with the risk of developing IgAN [29]. The latest GWAS [30] identified 30 susceptibility loci. This study noted a positive genetic correlation between a disease phenotype, IgAN, and a quantitative phenotype, serum levels of IgA, and determined a correlation between the total number of risk alleles and early disease onset. Some of those risk loci contain genes involved in mucosal innate immunity, such as *DEFA*, *VAV3*, *LIF*, *OSM*, and *TNFSF13*. *DEFA* encodes α-defensin, which is involved in mucosal innate immunity [31]. *VAV3* encodes a protein related to the NF-κB activation pathway [32]. Leukemia inhibitory factor (LIF) and oncostatin M (OSM) are cytokines of the IL-6 superfamily [33]. *TNFSF13* encodes APRIL, which promotes B-cell maturation and mucosal IgA class switching [20].

In addition to the genetic control of the production of IgA [34] and Gd-IgA1 [35,36], cytokines can also influence these phenotypes [37]. Specifically, IL-6 can induce the overproduction of Gd-IgA1 [19,38,39]. IL-6 can also upregulate *APRIL* and downregulate *C1GALT1*, the gene encoding the enzyme that adds Gal to IgA1 *O*-glycans. In addition to IL-6, LIF also induces aberrantly glycosylated IgA1 [40]. Notably, IL-6 and LIF mediate their activities through STAT signaling—STAT3 and STAT1, respectively. Importantly, the inhibition of NF-κB can reduce the production of aberrantly glycosylated IgA induced by IL-6 and TLR activation [25]. Thus, IL-6 and other cytokines, as well as NF-κB activation, might be involved in the pathogenesis of IgAN by enhancing the production of the main autoantigen.

## 6. Significance of Gd-IgA1-Containing Immune Complexes

Among several concepts regarding the pathogenesis of IgAN, the multi-hit theory has been widely accepted [12]. Human IgA1 has a hinge-region segment with nine serine/threonine residues, of which three to six are commonly glycosylated. Gd-IgA1 glycoforms are recognized by IgG autoantibodies to form immune complexes (ICs) [41]. IgG codeposits are not always detected in the kidney biopsy specimens of IgAN patients by routine immunofluorescence microscopy. However, a nanobody specific for the CH3 domain of the Fc portion of IgG detects IgG in all patients with IgAN [4,42].

Serum levels of Gd-IgA1 predict disease progression in patients with IgAN [43,44]. From the two studies that reviewed published data on Gd-IgA1, one concluded that the level of Gd-IgA1 in the serum or supernatant of cultured cells from the peripheral blood or tonsil is “likely to be a useful biomarker for the diagnosis of IgAN, though the Gd-IgA1 level does not appear to be associated with disease severity”, whereas the other study determined that serum Gd-IgA1 levels negatively correlated with kidney function [45,46]. Moreover, two studies that used glycan profiling by mass spectrometry found specific features of IgA1 *O*-glycosylation that correlated with kidney function in IgAN patients [47,48]. However, the main molecular forms of Gd-IgA1 that may be associated with clinical outcomes still need to be determined. Consequently, although the etiopathogenetic role of Gd-IgA1 has been demonstrated in IgAN, simple tests for the detection and quantification of Gd-IgA1 levels have not yet entered clinical practice. 

A fraction of Gd-IgA1 complexes can pass through the glomerular filter and be excreted in the urine, thus representing a possible disease-specific marker of IgAN [49]. Moreover, serum levels of IgA-IgG ICs correlate with the degree of hematuria and proteinuria [50]. These clinical findings suggest that ICs containing Gd-IgA1 and autoantibodies and other serum proteins, such as complement C3, are essential effector molecules in the pathogenesis of IgAN [51,52]. Other complement-regulating proteins, such as complement factor H (CFH) and CFH-related (CFHR) proteins 1-5, further modulate C3 processing and, thus, the biological activities of these Gd-IgA1-C3-IgG-containing immune complexes [53,54,55,56,57]. These complement-activating and complement-regulating pathways may provide new opportunities for therapeutical approaches [58,59].

## 7. Alternative Mechanisms Involved in the Development and Regulation of Glomerular Deposition of Nephritogenic IgA1-Containing Immune Complexes

The mechanism of glomerular deposition of IgA1-ICs is still not fully understood. Compared to uncomplexed IgA1, the clearance of high-molecular-mass ICs by the liver is reduced, increasing the likelihood of deposition in the glomeruli and driving the subsequent injury through complement activation [60]. In addition to the autoantibody-driven formation of IgA1-containing immune complexes, other possible mechanisms have been considered.

Cambier et al. found in IgAN patients circulating complexes formed from IgA1 and a soluble fragment of CD89 (sCD89-IgA complexes). Moreover, elevated levels of circulating sCD89-IgA complexes and free sCD89 were found in pediatric IgAN patients and were associated with proteinuria and MEST pathological scores [61]. These sCD89-IgA ICs interact in the glomeruli with transferrin receptors (TfR1) of mesangial cells.

In addition, Takahata et al. reported that the apoptosis inhibitor of macrophages (AIM, also known as CD5-like) protein is required to form inflammation-inducing glomerular immunodeposits in a mouse model of IgAN [62]. AIM-deficient gddY mice developed mesangial IgA immunodeposits but without co-deposits of IgG, IgM, and C3. Importantly, i.v. administration of recombinant AIM protein to these AIM-deficient gddY mice restored glomerular IgG, IgM, and C3 co-deposits. Furthermore, AIM was detected by immunofluorescence microscopy in patients with IgAN, and it was partially colocalized with IgA, IgG, and IgM in the glomerular immunodeposits. Conversely, AIM is not associated with IgA- or IgM-containing circulating immune complexes in gddY mice, indicating that AIM associates with the immunodeposits in situ. AIM protein is a cysteine-rich soluble scavenger receptor with pleiotropic roles in normal physiology as well as the pathophysiology of several diseases, and it contributes to the resolution of inflammatory conditions and the removal of cellular debris and aggregates [63]. Notably, AIM binds to the Fc segments of pentameric J chain-containing IgM through the formation of disulfide bonds [64], resulting in an extended time of AIM in circulation. Future studies are needed to determine whether AIM participates in the removal and clearance of mesangial immunodeposits and recovery from injury or whether it is part of a pathogenetic process in some patients with IgAN.

Regarding IgM and its role in IgAN, Matsumoto et al. found that some circulating ICs from patients with IgAN contain anti-Gd-IgA1 IgM antibodies and complement C3 [65]. These IgAM antibodies also recognized GalNAc in glycan-binding tests. This observation may explain glomerular IgM co-deposition in some patients with IgAN. In the early study of immune complexes and anti-Gd-IgA1 antibodies, it was shown that patients with IgAN had higher levels of IgG autoantibodies specific for Gd-IgA1 compared to healthy and disease controls [66]. Conversely, IgM from the sera of patients with IgAN and healthy controls exhibited similar binding to Gd-IgA1 [66]. Future studies should determine whether IgM specific for GalNAc that binds to Gd-IgA1 may be related to protective mechanisms represented by natural anti-glycan antibodies [67] or rather a part of the pathogenic process, and, furthermore, whether the role of AIM protein may be converging with that of these IgM antibodies.

Another possible mechanism explaining the deposition of IgA1 in glomerular mesangial was revealed by Nihei et al. [68]. The group identified an IgA autoantibody against mesangial cells in a gddY mouse model of IgAN [68]. Mass-spectrometry analysis identified that the autoantigen recognized by serum IgA from these mice is βII-spectrin and that this antigen is expressed on the cell surface of mesangial cells. The binding of serum IgA to βII-spectrin of mesangial cells is thus another possible mechanism for the mesangial IgA deposition. Many IgAN patients also appear to have serum anti-βII-spectrin IgA antibodies, and it was postulated that serum levels of these anti-βII-spectrin IgA antibodies represent a novel biomarker for IgAN [68]. A new study described the discovery of another autoantigen in gddY mice recognized by IgA antibodies induced by cross-reactive antigen from a specific strain of oral streptococci [69]. This new antigen, chromobox homolog 3 (CBX3), is a component of heterochromatin and also binds lamin B receptor, an integral membrane protein found in the inner nuclear membrane. CBX3 is overexpressed in human cancer [70]. CBX3 is presented on the cell surface of mesangial cells [69], where it is recognized by cross-reacting antibodies. However, the mechanism by which CBX3 is presented on the surface of mesangial cells is not known.

In summary, IgAN is known for the characteristic glomerular deposits of IgA1 that are enriched for Gd-IgA1 glycoforms. These immunodeposits are thought to originate from the circulating immune complexes formed from Gd-IgA1 and IgG autoantibodies specific for Gd-IgA1, with complement C3 added. Some of these circulating complexes deposit in the glomeruli and activate mesangial cells, thus inducing kidney injury. There may be additional ways for IgA deposits to develop, as detailed above. The roles of these various other components and pathways in human IgAN and their pathogenic potential remain to be further clarified. 

## 8. Conclusions

Accurate risk stratification of individuals at diagnosis and predicting treatment response in patients with IgAN remain challenging. Noninvasive and real-time examination using biomarkers directly related to the pathogenesis of the disease is critical to selecting a desired treatment protocol and assessing its effectiveness. Gd-IgA1-containing immune complexes are recognized as the key pathogenic component of the disease. Therefore, understanding mechanisms contributing to the production of Gd-IgA1 and the formation of the pathogenic immune complexes are important for the efforts to develop new therapeutic approaches and suitable biomarkers. As TLR9 and TLR7 play a crucial role in the overproduction of Gd-IgA1 and affect the disease progression of IgAN via IL-6/APRIL-mediated pathways, they are emerging as new possible therapeutic targets. The inhibition of TLR9/TLR7- and IL-6/APRIL-mediated pathways may provide new strategies for the future treatment of IgAN that would ultimately reduce the production of Gd-IgA1 and Gd-IgA1-containing immune complexes.
